# Association of high Body Mass Index and postdates pregnancy

**DOI:** 10.12669/pjms.38.5.4815

**Published:** 2022

**Authors:** Qudsia Qazi, Nazia Liaqat, Shehzadi Saima Hussain, Wajeeha Syed

**Affiliations:** 1 Dr. Qudsia Qazi, FCPS (Obs&Gynae) Associate Professor, Department of Obstetrics & Gynaecology, Medical Teaching Institute, Lady Reading Hospital, Peshawar, Pakistan; 2 Dr. Nazia Liaqat, FCPS (Obs&Gynae) Assistant Professor, Department of Obstetrics & Gynaecology, Medical Teaching Institute, Lady Reading Hospital, Peshawar, Pakistan; 3 Dr. Shehzadi Saima Hussain, FCPS (Obs&Gynae) Assistant Professor, Department of Obstetrics & Gynaecology, Medical Teaching Institute, Lady Reading Hospital, Peshawar, Pakistan; 4 Dr. Wajeeha Syed, FCPS (Obs&Gynae) Assistant Professor, Department of Obstetrics & Gynaecology, Medical Teaching Institute, Lady Reading Hospital, Peshawar, Pakistan

**Keywords:** Body mass index, Postdates pregnancy, Induction of labor, Caesarean section

## Abstract

**Background and Objective:**

Obesity with its growing prevalence is a major public health problem influencing gestational age at delivery. Raised Body Mass Index (BMI) has been shown to be associated with significantly increased risk of prolonged pregnancy; which is an important contributor to perinatal morbidity and mortality. Obesity needs modified antenatal, intrapartum and postpartum care by obstetrician. Limited data is found regarding association of obesity with prolonged pregnancy and the same fact led us to search for this association.

**Methods:**

This cohort study was carried out in Gynecology and Obstetrics department, MTI Lady Reading Hospital Peshawar from March 2020 to April 2021. Patients were enrolled in third trimester at 37 weeks of gestation with primary exposures of interest being either self-reported pre-pregnancy weight or obtained from first trimester antenatal record. Patients were divided into two classes based on BMI i.e., one with BMI <25 and other with BMI ≥ 25. Patients in both classes were followed till their delivery to determine outcome of gestational age at delivery.

**Results:**

Statistically significant difference between the two groups was seen at lower age range of 18-21 years(p-0.04) and higher age range of 39-42 years (p-0.0001). Statistically significant association was found between high pre pregnancy BMI and postdates pregnancy (OR: 4.93 ;95% CI: 1.98-12.26, p-0.001). Association of induction of labor with high pre pregnancy BMI was not significant. (OR 0.56, 95% CI: 0.21-1.48, P < 0.001). Higher rates of Instrumental deliveries(p-0.0005) and cesarean sections (p-0.0001) were seen in higher BMI group.

**Conclusion:**

Higher pre-pregnancy BMI is associated with increased risk of postdates pregnancy.

## INTRODUCTION

Maternal obesity i.e. pre-pregnancy body mass index (BMI) ≥29.9 kg/ m2)] is becoming a major public health problem. It can have grave consequences in pregnancy and needs serious consideration; necessitating modified care by obstetrician in antenatal, intrapartum and postpartum period.^1^

WHO and the National Institutes of Health define body mass index as weight in Kilogram/height in m^2^. The World Health Organization (WHO) has categorized BMI as:1) underweight i.e. BMI <18.5 kg/ m^2^ ,2) Recommended weight i.e. BMI 18.5–24.9 kg /m^2^ ,3) over weight with BMI, 25.0–29.9 kg/ m^2^, and 4) Obese with BMI of ≥30.0 kg /m^2^. Obesity is then further sub classified into three classes.^2^ For Asian populations, the BMI values are reduced (normal weight 18.5–23 kg/ m^2^, overweight 23–27.5 kg/ m^2^ and obese >27.5 kg /m^2^) because of increased risk of metabolic diseases at relatively lower BMI.^3^ However, this Asian specific criterion is not consistently adopted internationally in research or clinical guidelines.^4^

The WHO reports prevalence of obesity in pregnancy to be between 1.8% and 25.3% .^5^ In a Turkish study the prevalence of obesity and overweight in pregnancy was 48% .^6^ Increasing prevalence of obesity in both developed and developing world due to increased sedentary lifestyle, changes in diet and reluctance to implement public health policies to challenge obesity, have resulted in population different from that of 1950s.^7^

Adipose tissue produces and releases peptide hormones( leptin , steroid hormones) and adipokines (cytokines, adipsin and acylation-stimulating protein, adiponectin, resistin) which have dys-regulatory effect on many tissue functions including myometrial contractility leading to increased risk of postdates pregnancy,^3^altered quality of labor, caesarean section and postpartum hemorrhages.^8^ Several large studies found an increase in prolonged pregnancy (41 weeks or beyond) or post – term pregnancy (42 weeks and beyond) with increasing BMI .^9^

Self-reported approximation of gestational age relying on last menstrual period can over estimates post term pregnancy prevalence. The accuracy in estimation of gestational age has been tremendously improved by the advent and use of ultrasound in obstetrics. The primary predictor i.e pre-pregnancy weight has been validated however underestimation of pre-pregnancy weight by obese women will lead to an overestimation of weight gain during pregnancy .^10^,^11^

High BMI and post-dates pregnancy have been shown to increase the risk of maternal and neonatal adverse outcomes including pre-eclampsia, prolonged labor, cesarean section, fourth degree perineal lacerations, postpartum hemorrhage, fetal compromise, fetal dysmaturity, macrosomia, shoulder dystocia, perinatal mortality, birth injury, low APGAR scores, late fetal death, congenital malformations, meconium aspiration syndrome, and increased neonatal intensive care unit admissions.^1^,^11^ Patients trust their health care workers and rely on their information. Pre conception or early conception period is the most appropriate time to investigate obesity and find solution for it.^12^

There is limited literature available on the association of high BMI and postdates pregnancy from developing countries; where many medical complications have been proven to occur at lower BMI thresholds.^13^ The same fact led to search for association of raised pre pregnancy BMI with prolonged pregnancy, in our population. The findings of this study can be utilized in provision of evidence based information to our patients and to incorporate changes to their management in pre pregnancy, and pregnancy period.

## METHODS

This cohort study was carried out in gynecology and obstetrics department of Lady Reading Hospital Peshawar from March 2020 to April 2021. Formula used to calculate sample of 390 patients is n= Z2. P (1- P)/ d2. Ethical approval was obtained from institutional review board (Ref# 557, dated 07-08-2020).

### Inclusion and Exclusion criteria:

Patients included had single alive fetus with cephalic presentation, available data about their own weight in the first trimester of pregnancy, with gestational age of 37 weeks and low risk. Patients excluded were those who had no available data on ultra-sonography in first half of pregnancy, with fetal anomalies, preterm labor, medical complications in mother e.g. diabetes or chronic hypertension before pregnancy or who had previous history of postdates pregnancy. Informed consents were taken from all included patients. Non probability consecutive sampling was done. Evaluation of all patients was done by detailed history and clinical examination on admission. Basic demographics like age, parity, gestational age were noted. Booking BMI of all patients was determined from antenatal record. Records of patients were maintained on a proforma at gynae and obstetric unit of Lady Reading Hospital Peshawar. Early ultrasound scan (before 12 weeks of gestation) were used for estimation of gestational age. Patients were divided into two classes based on booking BMI i.e. one with BMI <25 and other with BMI ≥ 25. The women’s weight taken up to 12 weeks of gestation was considered her weight before pregnancy. Patients in both classes were followed from 37 weeks till their delivery to determine primary outcome of gestational age at delivery.

Data was analyzed using SPSS version 25. Mean and standard deviation were computed for continuous variables like age, gestational age, weight, height and BMI; Frequency and percentages were calculated for qualitative variables like parity and postdates pregnancy. A two tailed P value of <0.05 was considered to be significant. Univariate regression analysis was run to find out the strength of association for statistically significant associations of BMI with Post dates pregnancy, and BMI with induction of labor.

## RESULTS

The target population was 390 pregnant women. Out of total, women with BMI ≥ 25 were 135 and < 25 were 255. The mean age of the women was 28.2 ± 4.8 years. There is statistically significant difference (i.e. P value of 0.04) between two groups within age groups of 18-20 years as 12.5% of women were in BMI group of< 25 compared to 5.9% with ≥25.Similarly highly statistically significant difference existed between the two groups at age range of 39-42 years (p-0.0001) (Table-I).

**Table I:** Demography.

BMI	Age						

	18-21 yrs	22-25 yrs	26-29 yrs	30-34 yrs	35-38 yrs	39-42 yrs	Total
≥25 BMI	8(5.9%)	30(22.2%	30(22.2%)	27(20%)	28(20.7%)	12(8.9%)	135(34.6%)
<25BMI	32(12.5%)	71(27.8%)	51(20.0%)	62(24.3%)	36(14.1%)	3(1.2%)	255(65.4%)
Total	40(10.2%)	101(25.8%)	81(20.7%)	89(22.8%)	64(16.4%)	15(3.8%)	390(100%)
p-Value	0.04	0.22	0.26	0.33	0.09	0.0001	

Association of pre-pregnancy BMI and gestational age at delivery is shown in Table-II. Significant association of postdates pregnancy with high BMI was found. (OR:4.93, 95%CI:1.98-12.26, p value= 0.001).

**Table II:** BMI and Gestational Age at Delivery.

BMI	Gestational Age			

	37-39 +6(Wks)	40-41(Wks)	Total	p value
<25	159 (63%)	96 (37%)	255	<0.001
≥25	36 (27%)	99 (73%)	135	< 0.001

Frequency of induced and spontaneous delivery based on BMI is shown in Table-III. Association of BMI with induction of labor was not significant, with an OR of 0.56 (95%CI:0.21-1.48), p-0.001.

**Table III:** BMI and induction of labor.

BMI	Induction		

	Spontaneous	Induced	Total
<25	235(92.2%)	20(7.8%)	255(65.4%)
≥25	82(60.7%)	53(39.3%)	135(36.6%)
Total	317(81.3%)	73(18.7%)	390(100%)
p value	<0.001	<0.001	

The differences in the modes of deliveries between the two groups are shown in Table-IV. Statistically significant differences were seen between the two groups across all modes of deliveries, Normal vaginal delivery being higher in BMI<25 (p-0.0001). Instrumental deliveries were higher in BMI ≥25 (p-0.0005). Similarly, statistically significant difference was seen in the rates of cesarean deliveries between the two groups, cesarean rates being higher for BMI ≥25(p-0.0001)

**Table IV:** BMI and Mode of Delivery.

BMI	Mode of Delivery			

	Normal vaginal delivery	Instrumental delivery	Cesarean section	Total
<25	239 (61.3%)	8 (2.1%)	8 (2.1%)	255 (65.4%)
≥25 BMI	77 (19.7%)	19 (4.9%)	39 (10.0%)	135 (34.6%)
TOTAL	316 (81.0%)	27 (6.9%)	47(12.1%)	390 (100%)
p-values	0.0001	0.0005	0.0001	

## DISCUSSION

The present study proves the association of pre pregnancy BMI with postdates pregnancy. The global pandemic of overweight and obesity has led to growing number of pregnant women with high BMI. Maternal pre-pregnancy BMI and gestational weight gain, both have immediate and long-term health implications for mother and offspring.^13^ Careful preemptive strategies are required for reducing weight prior to conception in order to avoid unintended consequences.

In our study 70.3% of women of BMI> 25 were within age groups of 18-34 years, while 84.6% women of BMI < 25 were in this age range; which is comparable to findings of a study by Shama Munim with 90.7% women having age between 19-35 years.^13^

In the present study, 73% of women with higher pre-pregnancy BMI delivered at 40-41 weeks of gestation (post-dates pregnancy) compared to 37% of those with normal BMI. Various studies have confirmed this relationship as Halloran found an adjusted odds ratio of 1.21 and 1.27 respectively for postdates pregnancy in women having gestational weight gain within or above recommendations.^14^,^15^

A Scottish study found no association between obesity and postdate pregnancy (aOR 1.47, 95% CI: 0.78 to 2.77).^16^ A study by Emma found that absolute risk of post-term birth increased monotonically as BMI category increased (4.9, 6.2, 6.9, 7.2, 8.1, 8.4, and 9.9 )for underweight, recommended, over weight and obese class: I, II, IIIa and IIIb respectively, with an adjusted OR 2.80, 95% CI 1.31-5.98.^17^ Usha Kiran also observed an increased risk (with a quoted OR of 1.4) of postdates pregnancy in women with increasing booking visit BMI measured by midwives.^18^

Interruption of the natural gestational trajectory with interventions to expedite birth, such as induction of labor and cesarean section are challenges of maternal obesity and post-term pregnancy.^19^ The present study shows association between pre-pregnancy BMI and type of delivery i.e. an increase in need for IOL and cesarean section with higher BMI, however the association of induction of labor in our study is not significant. Regarding induction of labor, Usha found significant association (OR 1.28, 95% CI: 1.23 to 1.33) and (OR 1.69, 95% CI: 1.62 to 1.76) for overweight and obese women respectively, as compared to normal weight women.^18^ Doi observed higher ORs of 1.80 (95% CI: 1.73 to 1.88) and 3.14 (95% CI: 3.00 to 3.29) for elective and emergency caesarean sections respectively in obese women compared to normal weight women.^16^ A study by Usha Kiran showed comparable results with OR of 1.6 for induction of labor.^18^

In randomized controlled studies, by adding diet and exercise to behavioral interventions before pregnancy, BMI was reduced in 12-month follow up and it was shown that decreasing BMI by increasing physical activity positively affected the prognosis of pregnancy.^20^

This study has found a stronger association between high BMI and postdate pregnancy. The findings of this study highlight the need for nutritional education and life style modification to improve pregnancy outcomes. BMI before pregnancy directly affects weight gain during pregnancy and the importance of pre-pregnancy counseling and weight loss is emphasized once again. Limited literature is available on the pattern of pregnancy weight gain of women from developing countries. Hence, there is a need to see the contextual relevance of these recommendations to our own population. In some studies, it was found that pregnant women who were recommended weight gain according to IOM guidelines had a higher probability of gaining appropriate weight than those who were not given recommendations.^10^ In addition to giving education and losing weight before pregnancy, the importance of personalized diet, exercise, and nutrition education during pregnancy is clear. Implementation of diet, exercise, and training at the same time is important.

### Limitations of the study:

The present study was a single centered study with small sample size. The use of booking weight as an indicator of pre-pregnancy weight is one of the limitation of this study. This one is a widely used method, since practically it is not possible for every woman to know how much they weight, right before their pregnancy. However, it is a prospective observational study as compared to many retrospective studies done previously.

## CONCLUSION

Elevated pre-pregnancy BMI increase the risk of postdate pregnancy.

### Recommendation:

Future maternal obesity research should consider the heterogeneity between obesity classes. Health professionals should be empowered and trained to deliver promising dietary and lifestyle interventions to women at risk of overweight and obesity prior to conception, and control excessive weight gain in pregnancy.

### Author`s Contribution:

**QQ:** Conceived the idea, collected data, drafted manuscript.

**NL:** Statistical analysis, edited manuscript, finalized manuscript.

**SSH:** Data collection, revised manuscript.

**WS:** Data collection, preparing the manuscript

All authors share responsibility for integrity and accuracy of work.
